# P-1135. Influence of Antibiotic and Laxative Use on *Clostridioides difficile* Testing in Post-Operative Children

**DOI:** 10.1093/ofid/ofae631.1322

**Published:** 2025-01-29

**Authors:** Ruchita Negi, Abhishek Deshpande, Sarah Worley, Venkatraman Arakoni, Charles B Foster

**Affiliations:** Children's Specialty Group, Norfolk, Virginia; Cleveland Clinic, Cleveland, Ohio; Cleveland Clinic, Cleveland, Ohio; Cleveland Clinic, Cleveland, Ohio; Cleveland Clinic Children's, Cleveland, Ohio

## Abstract

**Background:**

Antibiotic exposure in surgical patients poses risks, contributing both to *Clostridioides difficile* infection (CDI) and to antibiotic associated diarrhea. While CDI may cause diarrhea, we hypothesized that negative CDT could be a proxy for non-infectious diarrhea and aimed to discern if broad-spectrum (BS) antibiotics or laxatives influenced negative CDT.

Broad Spectrum Antibiotics and Laxative Use In Post-Operative Children with Negative C difficile Test Compared to Matched Controls

**Figure:**
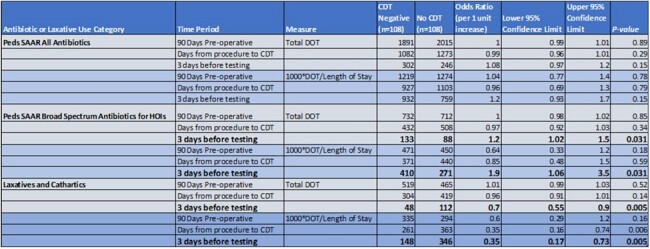

CDT: C difficile Testing, DOT: Days of Therapy, SAAR: Standardized Antibiotic Administration Ratio

**Methods:**

A matched case-control study (Jan 2013-Feb 2022) examined risk factors for negative CDT in children (2-18 years) undergoing sedated procedures with preoperative antibiotic prophylaxis at a large referral hospital. Perioperative data was merged with antibiotic and laxative data from the Electronic Medication Administration Record (eMAR). Cases with PCR-negative CDT on postoperative days 3-90 were matched with untested controls based on age, gender, year of testing, and procedure category. We assessed Days of Therapy (DOT) of antibiotics or laxatives for various periods.

**Results:**

Among 14,229 children (mean age 10 ± 5.2 years) undergoing 18,705 procedures, 108 cases and 108 controls were selected. Children with negative CDT received more BS antibiotics 3 days before the test date (1000DOT/LOS = 410) than controls (1000DOT/LOS = 271; OR 1.9 (1.06 – 3.5); P =0.031). Conversely, cases received fewer laxatives postoperatively and 3 days before CDT (1000DOT/LOS =148) than controls (1000DOT/LOS = 346; OR 0.35 (0.17-0.73); P=0.005). No difference was observed in pre-operative laxative use.

**Conclusion:**

Our data suggests that BS antibiotics are a more important contributor to post-operative non-infectious diarrhea in children than laxatives. Antimicrobial stewardship efforts targeting BS antibiotic use in post-operative children could mitigate diarrhea and unnecessary CDT.

**Disclosures:**

**Abhishek Deshpande, MD, PhD**, AHRQ: Grant/Research Support|Clorox Healthcare: Grant/Research Support

